# Draft genome sequences for ten strains of *Xanthomonas* species that have phylogenomic importance

**DOI:** 10.1099/acmi.0.000532.v3

**Published:** 2023-07-14

**Authors:** Jamie Harrison, Rana Muhammad Fraz Hussain, Shannon F. Greer, Vardis Ntoukakis, Andrew Aspin, Joana G. Vicente, Murray Grant, David J. Studholme

**Affiliations:** ^1^​ Biosciences, University of Exeter, Exeter, EX4 4QD, UK; ^2^​ Gibbet Hill Campus, School of Life Sciences, University of Warwick, Coventry, CV4 7AL, UK; ^3^​ Wellesbourne Campus, School of Life Sciences, University of Warwick, Coventry, CV35 9EF, UK; ^4^​ Fera Science Ltd., York Biotech Campus, Sand Hutton, York, YO41 1LZ, UK

**Keywords:** genome sequencing, phylogenomics, *Xanthomonas*, taxonomy

## Abstract

Here we report draft-quality genome sequences for pathotype strains of eight plant-pathogenic bacterial pathovars: *Xanthomonas campestris* pv. *asclepiadis*, *X. campestris* pv. *cannae*, *X. campestris* pv. *esculenti*, *X. campestris* pv. *nigromaculans*, *X. campestris* pv. *parthenii*, *X. campestris* pv. *phormiicola*, *X. campestris* pv. *zinniae* and *X. dyei* pv. *eucalypti* (= *X. campestris* pv. *eucalypti*). We also sequenced the type strain of species *X. melonis* and the unclassified *Xanthomonas* strain NCPPB 1067. These data will be useful for phylogenomic and taxonomic studies, filling some important gaps in sequence coverage of *Xanthomonas* phylogenetic diversity. We include representatives of previously under-sequenced pathovars and species-level clades. Furthermore, these genome sequences may be useful in elucidating the molecular basis for important phenotypes, such as biosynthesis of coronatine-related toxins and degradation of fungal toxin cercosporin.

## Data Summary

Protocols used in this study are deposited at protocols.io:

- Culturing *Xanthomonas* strains: https://dx.doi.org/10.17504/protocols.io.ewov1nr92gr2/v1.
- Preparing genomic DNA: https://dx.doi.org/10.17504/protocols.io.5jyl89428v2w/v1.
- Genome sequence assembly from Illumina reads: https://dx.doi.org/10.17504/protocols.io.kxygxzrqzv8j/v1.
- Phylogenomic analysis: https://dx.doi.org/10.17504/protocols.io.261geny57g47/v1.

The sequence data described in this article are available under BioProject accession PRJNA774146 via https://www.ncbi.nlm.nih.gov/bioproject/PRJNA774146/. Accession numbers of individual genome assemblies are listed in Table 1. Configuration files and tree files are available from GitHub at https://github.com/davidjstudholme/phylogenomics-Xanthomonas-1.

## Introduction

The genus *Xanthomonas* is comprised of Gram-negative bacteria that are usually associated with plants and it includes causative agents of economically important disease of crops such as brassicas, rice, cassava, banana and tomatoes [1]. The taxonomy of *Xanthomonas* spp. has a long and sometimes confusing history. The List of Prokaryotic names with Standing in Nomenclature (accessed 3 April 2023) lists 35 validly named species for the genus, plus several synonyms and others that have not been validly published [2]. Many *Xanthomonas* species are further divided into intra-specific groups called pathovars, which are defined primarily by their host range and in some cases also by biochemical or physiological differences [3].

Recent efforts have attempted to reconcile the taxonomy of *Xanthomonas* species and pathovars with their phylogeny, often informed by sequence data from one or several genes or from genome-wide sequence data; this has led to the proposal of new species [4–8] and the transfer of pathovars from one species to another [9–19]. Analysis of partial DNA sequences of the *gyrB* gene suggested that many pathovars of the species *X. campestris* are phylogenetically not closely related to the type strain of this species; they are much closer to other named species or so-called species-level clades (slc) [20].

Although partial *gyrB* gene sequences are available for many of these taxonomically incongruent strains, genome-wide sequence data would facilitate phylogenomic studies at a higher resolution. Here, we present draft-quality genome sequences for strains that nominally belong to *X. campestris *, but whose inclusion in that species is incongruent with phylogeny [6, 20–23]; evolutionarily, they fall within species ‘*X. cannabis’*, *X. dyei *, *X. hortorum *, *X. melonis *, *X. sacchari *, Slc 4 and Slc 6. Furthermore, we sequenced strain NCPPB 4013 [24], representing a putative new species, and the type strain of *X. melonis *, thereby filling some gaps in the resources for phylogenomics of *Xanthomonas *.

## Methods

Bacterial strains were obtained from the National Collection of Plant Pathogenic Bacteria (https://www.fera.co.uk/ncppb). We cultured the bacteria in King’s B liquid medium at 28 °C as described in protocols.io at https://dx.doi.org/10.17504/protocols.io.ewov1nr92gr2/v1. Genomic DNA was extracted from frozen bacterial pellets using the Qiagen 67 563 MagAttract HMW DNA Kit, following protocol described at https://dx.doi.org/10.17504/protocols.io.5jyl89428v2w/v1. Whole-genome shotgun sequencing was performed on the Illumina NovaSeq 6000 platform to generate pairs of 151-nucleotide sequence reads. We subjected the resulting sequence reads to quality control with fastp [25], *de novo* assembly with SPAdes version 3.15.1 [26] and polishing with Pilon [27] as documented in protocols.io at https://www.protocols.io/view/de-novo-assembly-of-xanthomonas-genomes-from-illum-kxygxzrqzv8j/v1. We generated a phylogenomic tree from genome sequence assemblies using PhaME [28] and FastTree [29] as described in the protocol at https://protocols.io/view/phylogenomic-analysis-of-xanthomonas-ces2tege. Average nucleotide identities (ANI) were calculated using FastANI [30] as described in the protocol at https://www.protocols.io/view/fastani-analysis-protocol-261ge36bol47/v1. For calculating dDDH values, we used the Type Strain Genome Server [31], using formula two (sum of all identities found in high-scoring pairs [HSP] divided by total HSP length) [32]. Genome assemblies were submitted to GenBank [33] via the NCBI Submission Portal [34], after which they were annotated via the NCBI Prokaryotic Genome Annotation Pipeline (PGAP) version 5.3 [35]. Assembly quality was assessed using CheckM version 1.2.2 [36].

## Results and discussion

We assembled draft genome sequences *de novo* from short sequence reads for ten strains of *Xanthomonas* species and deposited these in GenBank under the accession numbers listed in Table 1. These strains include the type strain for *X. melonis *, pathotype strains for eight pathovars of *X. campestris* and one strain (from radish) not assigned to any named species. For nine of the ten newly sequenced genomes, partial *gyrB* sequences are available under GenBank accessions EU007531.1, EU285211.1, EU285210.1, EU285202.1, EU285197.1, EU285181.1, EU285180.1, EU285177.1 and EU285064.1. In all cases, blastn searches confirmed 100 % nucleotide sequence identity over the full length of the partial *gyrB* gene versus the corresponding genome sequence assembly. CheckM estimated contamination levels at below 5 % for all assemblies except for that of *X. campestris* pv. *nigromaculans* ([Table T1] Tables 1 and S1), available in the online version of this article. Pairwise ANI values are tabulated in Table S2.

**Table 1. T1:** Bacterial strains used for genome sequencing. Contamination levels were assessed using CheckM [36]. Additional assembly metrics are provided in the Table S1

Taxonomic name	NCPPB no.	Host of isolation	Phylogenetic position	Genome assembly GenBank accession	Coverage	Contamination (%)
* X. campestris* pv. *esculenti* (Rangaswami & Easwaran 1962) Dye 1978	2190 (PT)	*Hibiscus esculentus*	‘*X. cannabis*’ (Slc 1)	GCA_020784125.1	141 x	0.25
* X. campestris* pv. *zinniae* [42] Dye 1978	2439 (PT)	*Zinnia elegans*	‘*X. cannabis*’ (Slc 1)	GCA_020783815.1	134 x	1.12
* X. dyei* pv. *eucalypti* [12, 50] (= *X. campestris* pv. *eucalypti*)	2337 (PT)	*Eucalyptus maculata var*. *citriodora*	* X. dyei *	GCA_020783675.1	63 x	0.58
* X. melonis* [16]	3434 (T)	*Cucumis melo*	* X. melonis *	GCA_020783655.1	169 x	0.70
*X*nthomonas sp.	1067	*Raphanus sativus*	* X. melonis *	GCA_020783795.1	32 x	0.37
* X. campestris* pv. *parthenii* [54]	3888 (PT)	*Parthenium hysterophorus*	Slc 4	GCA_020783765.1	192 x	1.08
* X. campestris* pv. *phormiicola* [55] Dye 1978	2983 (PT)	*Phormium tenax*	Slc 6	GCA_020783715.1	49 x	4.484
* X. campestris* pv. *nigromaculans* [22] Dye 1978	1935 (PT)	*Arctium lappa*	* X. hortorum *	GCA_020783725.1	57 x	12.92
* X. campestris* pv. *cannae* [65]	4345 (PT)	*Canna*×*generalis*	* X. sacchari *	GCA_020783755.1	122 x	4.24
* X. campestris* pv. *asclepiadis* [24]	4013 (PT)	*Asclepias syriaca*	Close to *X. euroxanthea *	GCA_020784175.1	180 x	0.89

PT, Pathotype strain; T, Type strain.

### ‘*X. cannabis’*

The species ‘*X. cannabis*’ was proposed to include *X. campestris* pv. *cannabis*, and *X*. *sp*. strain Nyagatare isolated from beans [37, 38], though according to The List of Prokaryotic names with Standing in Nomenclature [2] (accessed 29 March 2023) this name is not yet validly published. Originally, *X. cannabis* was proposed in 1955 when Okabe and Goto transferred *Pseudomonas cannabis* [39] into the genus *Xanthomonas* [40]; however, no type strain was deposited. In 2014, Netsu and colleagues [41] speculated that a Japanese isolate identified as *X. campestris* pv. *cannabis* might represent the same pathogen [39] previously described as ‘*P. cannabis*’ and ‘*X. cannabis*’.

**Fig. 1.**
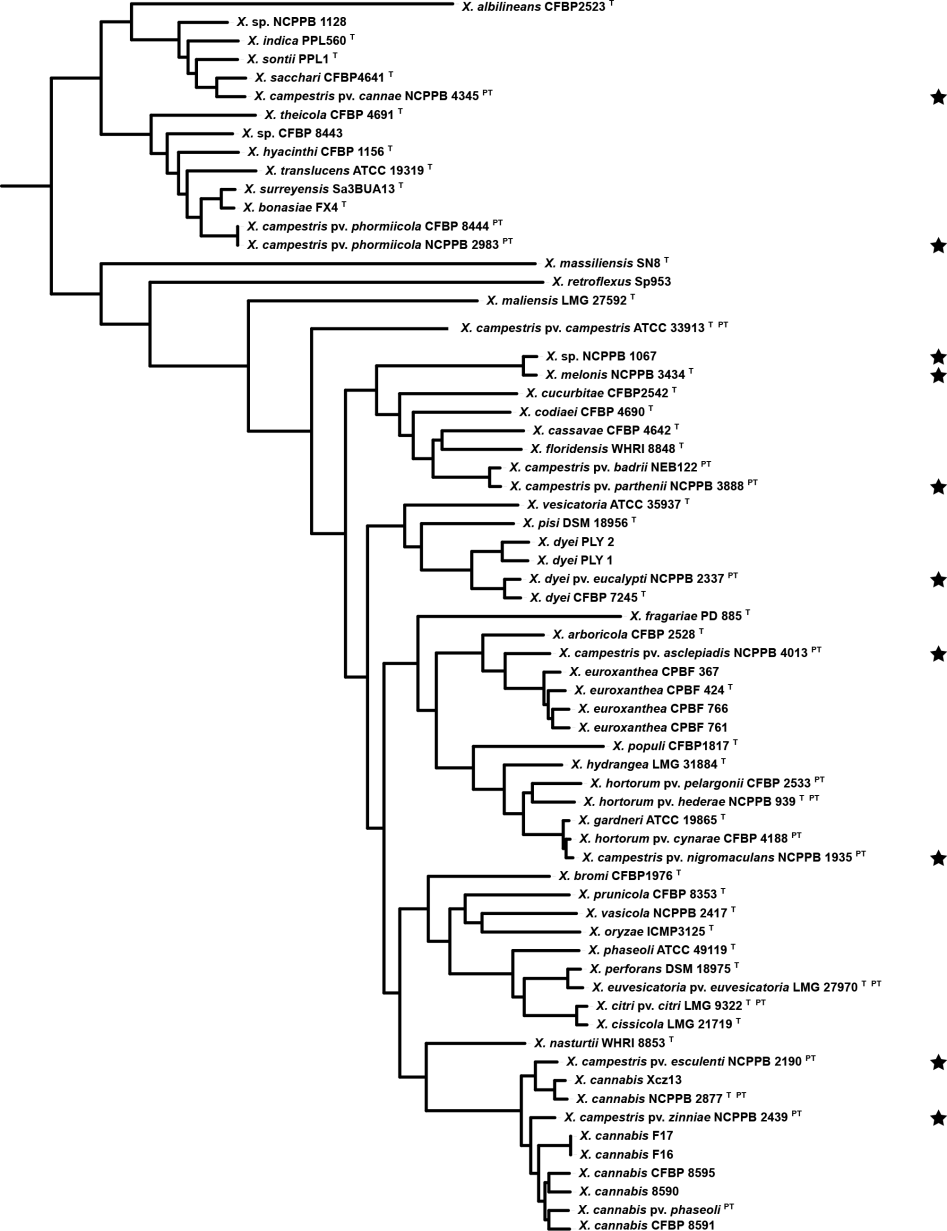
Phylogenetic tree, based on core-genome sequences, for the newly sequenced strains, type strains and representative strains of *Xanthomonas* spp., generated using PhaME [28] and FastTree [29]. The tree was graphically rendered using the Interactive Tree of Life [67]. Configuration and tree files are available from https://github.com/davidjstudholme/phylogenomics-Xanthomonas-1. Accession numbers and references for the genome assemblies are listed in Table 2. Newly sequenced genomes are indicated with a black star.

According to Parkinson [20], *X. campestris* pv. *cannabis* falls within Slc 1, along with pathotype strains of *X. campestris* pv. *zinniae* (NCPPB 2439) and *X. campestris* pv. *esculenti* (NCPPB 2190), originally ‘*X. rubrisorghi*’ [20]. This inclusion was based on partial *gyrB* gene sequences; genomes had not been previously sequenced. Our sequencing and phylogenomic analysis (Fig. 1, Table 2) confirm the close relationship between these pathovars and *X. campestris* pv. *cannabis*. Furthermore, they share 96.58 % and 97.67 % ANI (70.6 % and 79.4 % dDDH) with the proposed strain NCPPB 2877, consistent with their belonging to the same species.

**Table 2.** Genome assemblies used for phylogenomic analysis

GenBank accession	Bacterial strain	References
GCA_002939705.1	* X. albilineans* CFBP2523 ^T^	–
GCA_001013475.1	* X. arboricola* pv. *juglandis* CFBP 2528 ^T PT^	[68]
GCA_017163705.1	* X. bonasiae* FX4 ^T^	[4]
GCA_002939755.1	* X. bromi* CFBP1976 ^T^	[69]
GCA_020784175.1	* X. campestris* pv. *asclepiadis* NCPPB 4013 ^PT^	This study
GCA_012848175.1	* X. campestris* pv. *badrii* NEB122 ^PT^	–
GCA_000007145.1	* X. campestris* pv. *campestris* ATCC 33913 ^T PT^	[70]
GCA_020783755.1	* X. campestris* pv. *cannae* NCPPB 4345 ^PT^	This study
GCA_020784125.1	* X. campestris* pv. *esculenti* NCPPB 2190 ^PT^	This study
GCA_020783725.1	* X. campestris* pv. *nigromaculans* NCPPB 1935 ^PT^	This study
GCA_938743425.1	* X. campestris* pv. *nigromaculans* NCPPB 1935 ^PT^	[21]
GCA_020783765.1	* X. campestris* pv. *parthenii* NCPPB 3888 ^PT^	This study
GCA_025666215.1	* X. campestris* pv. *phormiicola* CFBP 8444 ^PT^	[56]
GCA_020783715.1	* X. campestris* pv. *phormiicola* NCPPB 2983 ^PT^	This study
GCA_020783815.1	* X. campestris* pv. *zinniae* NCPPB 2439 ^PT^	This study
GCA_011761725.1	*X. cannabis* 8590	–
GCA_014195785.1	*X. cannabis* CFBP 8591	–
GCA_014195835.1	*X. cannabis* CFBP 8595	–
GCA_014199075.1	*X. cannabis* F16	–
GCA_014199515.1	*X. cannabis* F17	–
GCA_000802365.1	*X. cannabis* NCPPB 2877 ^T PT^	[38]
GCA_000764855.1	*X. cannabis* pv. *phaseoli* Nyagatare ^PT^	[37]
GCA_020880755.1	*X. cannabis* Xcz13	–
GCA_000454545.1	* X. cassavae* CFBP 4642 ^T^	[71]
GCA_002019225.1	* X. cissicola* LMG 21719 ^T^	[18]
GCA_002018575.1	* X. citri* pv. *citri* LMG 9322 ^T PT^	[72]
GCA_002939785.1	* X. codiaei* CFBP 4690	–
GCA_002939885.1	* X. cucurbitae* CFBP2542 ^T^	–
GCA_002939865.1	* X. dyei* CFBP 7245 ^T^	–
GCA_003363885.1	* X. dyei* PLY_1	[73]
GCA_003363875.1	* X. dyei* PLY_2	[73]
GCA_020783675.1	* X. dyei* pv. *eucalypti* NCPPB 2337 ^PT^	This study
GCA_903989455.1	* X. euroxanthea* CPBF 367	[74]
GCA_900476395.1	* X. euroxanthea* CPBF 424 ^T^	[75, 76]
GCA_905367725.1	* X. euroxanthea* CPBF 761	[76]
GCA_905367735.1	* X. euroxanthea* CPBF 766	[76]
GCA_001401555.1	* X. euvesicatoria* pv. *euvesicatoria* LMG 27970 ^T PT^	–
GCA_001642575.1	* X. floridensis* WHRI 8848 ^T^	[8]
GCA_900380235.1	* X. fragariae* PD 885 ^T^	[77]
GCA_000192065.2	* X. gardneri* ATCC 19865 ^T^	[78]
GCA_021352995.1	* X. hortorum* pv. *cynarae* CFBP 4188 ^PT^	[79]
GCA_003064105.1	* X. hortorum* pv. *hederae* NCPPB 939 ^T PT^	[80]
GCA_021353095.1	* X. hortorum* pv. *pelargonii* CFBP 2533 ^PT^	–
GCA_009769165.1	* X. hyacinthi* CFBP 1156 ^T^	[81]
GCA_905142475.1	*X. hydrangea* LMG 31884 ^T^	[6]
GCA_022669045.1	*X. indica* PPL560 ^T^	[5]
GCA_009192945.1	* X. maliensis* LMG 27592 ^T^	[82]
GCA_900018785.1	*X. massiliensis* SN8 ^T^	[83]
GCA_020783655.1	* X. melonis* NCPPB 3434 ^T^	This study
GCA_001660815.1	* X. nasturtii* WHRI 8853 ^T^	[8]
GCA_004136375.1	* X. oryzae* ICMP3125 ^T^	[84]
GCA_013112235.1	* X. perforans* DSM 18975 ^T^	–
GCA_022749655.1	* X. phaseoli* ATCC 49119 ^T^	–
GCA_001010415.1	* X. pisi* DSM 18956 ^T^	[85]
GCA_002940065.1	* X. populi* CFBP1817 ^T^	–
GCA_002846205.1	* X. prunicola* CFBP 8353 ^T^	[86]
GCA_900143175.1	*X. retroflexus* Sp953	[87]
GCA_002940085.1	* X. sacchari* CFBP4641 ^T^	[7]
GCA_008119715.1	*X. sontii* PPL1 ^T^	[7]
GCA_020783795.1	* Xanthomonas* sp. NCPPB 1067	This study
GCA_014836395.1	*X. surreyensis* Sa3BUA13 ^T^	[88]
GCA_014236795.1	* X. theicola* CFBP 4691 ^T^	[89]
GCA_020880735.1	* X. translucens* pv. *translucens* ATCC 19319 ^T PT^	[90]
GCA_000772705.2	* X. vasicola* NCPPB 2417 ^T^	[19]
GCA_001908725.1	* X. vesicatoria* ATCC 35937 ^T^	[91]
GCA_025666195.1	* Xanthomonas* sp. CFBP 8443	[56]
GCA_025666235.1	* Xanthomonas* sp. CFBP 8445	[56]
GCA_001043115.1	* Xanthomonas* sp. NCPPB 1128	[92]

PT, Pathotype strain; T, Type strain.

A description of leaf spot on *Zinnia* plants in Zimbabwe [42] proposed the causative pathogen to be a *forma specialis* of *X. nigromaculans*, where *X. nigromaculans* was the pathogen responsible for leafspot on burdock [22] now known as *X. campestris* pv. *nigromaculans* and phylogenetically falling within *X. hortorum *. Subsequently, this *Zinnia* pathogen was renamed *X. campestris* pv. *zinniae* [3]. Previous analyses based on the *gyrB* locus [20] and our phylogenomic tree (Fig. 1) both place the *Zinnia* pathogen as completely distinct from *X. campestris* pv. *nigromaculans* and *X. hortorum *. Rather, it falls within the clade corresponding to ‘*X. cannabis*’ and Slc 1.

In addition to its significance as a pathogen in Africa [42], Asia [43, 44] and Europe [45], *X. campestris* pv. *zinniae* is of interest for its ability to degrade the toxin cercosporin that is produced by phytopathogenic fungi of the genus *Cercosporus* [46–49]. The search for genes encoding the degradation pathway identified an oxidoreductase and a putative transcriptional regulator but also highlighted that additional factors are required for cercosporin degradation [49]. Availability of this draft genome sequence may enable identification of additional genes that could facilitate the engineering of *Cercosporus*-resistant crop plants [49].

### X. dyei

Strain NCPPB 2337 causes dieback on *Eucalyptus citriodora* in Australia and was originally described as a new species: *X. eucalypti* [50]. In the major taxonomic revision that saw many species reclassified as pathovars of *X. campestris *, this pathogen was renamed as *X. campestris* pv. *eucalypti* [3], with NCPPB 2337 designated as the pathotype strain. In Parkinson and colleagues’ *gyrB*-based phylogenetic analysis [20], it was placed within Slc 2 along with unclassified xanthomonads isolated from *Lobelia* spp. and with *X. campestris* pv. *laureliae* isolated from *Laurelia novae-zelandiae*. Following multi-locus sequence analysis (MLSA), this slc was assigned the species name *X. dyei *, so that NCPPB 2337 became the pathotype strain of *X. dyei* pv. *eucalypti* [12]. Consistent with this, our phylogenomic analysis places *X. dyei* pv. *eucalypti* close to the type strain of *X. dyei* (Fig. 1), which was isolated from *Metrosideros excelsa* [12]. Values of ANI and dDDH were 96.86 % and 93.9 %, respectively.

### X. melonis

We sequenced the genome of NCPPB 3434 (=LMG 8670=CFBP 4644), type strain for species *X. melonis* [16], isolated from *Cucumis melo* in Brazil. This augments the previously available genome sequence for *X. melonis* CFBP 4644 (GenBank: GCA_002940015.1), and several recently sequenced isolates from Trinidad [51]. Strain CFBP 4644 is identical to NCPPB 3434 but it was not flagged as being type material in the metadata of BioSample SAMN05560295. Consistent with previous phylogenomic analyses of *X. melonis* CFBP 4644 [52, 53], the phylogenetic position of NCPPB 3434 fell within a clade shared with *X. cucurbitae *, *X. codiaei* and *X. floridensis* (Fig. 1).

*Xanthomonas* strain NCPPB 1067 was isolated from *Raphanus sativus* (radish) in Vanuatu. In the previous *gyrB*-based phylogenetic analysis [20], it was the sole representative of Slc 3. Our phylogenomic analysis places it close to the type strain of *X. melonis* (Fig. 1), with which it shares 98.57 % ANI and 86.80 % dDDH, consistent with its belonging to this species and with Parkinson’s Slc corresponding to the species *X. melonis *.

### *X. campestris* pv. *parthenii*: a potential new species (Slc 4)

*Xanthomonas campestris* pv. *parthenii* NCPPB 3880 causes leaf blight on *Parthenium*, an obnoxious exotic weed introduced into India in 1955, and might have potential as a biological control agent [54]. The pathotype strain NCPPB 3880 fell within Slc 4 along with *X. campestris* pv. *badrii* according to previous *gyrB* analysis [20]. In our phylogenomic analysis (Fig. 1), *X. campestris* pv. *parthenii* and *X. campestris* pv. *badrii* are closely related to each other, sharing 98.88 % ANI and 89.6 % dDDH. They appear as a distinct species neighbouring *X. floridensis* (93.66–93.68 % ANI and 52.2 % dDDH) and *X. cassavae* (93.31–93.35 % ANI and 50.3–50.7 % dDDH).

### *X. campestris* pv. *phormiicola*: a potential new species (Slc 6)

In 1933, Takimoto [55] described *Bacterium phormicola* as the cause of bacterial streak on New Zealand flax (*Phormium tenax*). This pathogen was subsequently classified as *X. campestris* pv. *phormiicola* [3], though Parkinson and colleagues’ later examination of *gyrB* sequences [20] placed it as the sole member of Slc 6, outside of *X. campestris *. Our genome-based phylogenetic analysis of NCPPB 2983 confirmed this (Fig. 1) and placed it as a distinct species close to *X. hyacinthi* and *X. translucens* [56]. It shares 95 % ANI with *X. bonasiae* FX4 (Table S2), which is on the borderline of the commonly used criterion for species delimitation. Based on dDDH values, the Type Strain Genome Server identifies that this genome does not fall within any named species and potentially represents a new species dDDH between NCPPB 2983 and *X. bonasiae* FX4 is 56.1. The recent study by Peduzzi and colleagues came to a similar conclusion [56].

*Xanthomonas campestris* pv. *phormiicola* is unusual among xanthomonads in that it produces the phytotoxin coronatine [57] and/or related derivatives of coronofacic acid [49], a trait more usually associated with *Pseudomonas* spp. Tamura and colleagues [57] hypothesized that phytotoxin biosynthesis might be encoded on a plasmid but failed to isolate plasmid DNA. The availability of genome sequence opens the possibility of identifying the genetic basis for this trait. After our submission of the first version of this manuscript, a high-quality genome sequence assembly was published for *Xanthomonas campestris* pv. *phormiicola* and candidate biosynthesis genes were identified [56].

### *X. campestris* pv. *asclepiadis*: a potential new species

Bacterial blight of milkweed (*Asclepias spp*.) is attributed to *X. campestris* pv. *asclepiadis* [24]. No sequence data was available for this pathogen in GenBank and therefore its phylogenetic position within the genus was unclear. Our analysis places the pathotype strain NCPPB 4013 very distant from the type strain of *X. campestris *, and closer to *X. euroxanthea* (Fig. 1), a species isolated from walnut trees (*Juglans regia*) [58]. The ANI between NCPPB 4013 and the *X. euroxanthea* type strain is 95.97 %, close to the threshold of 95–96 % that is commonly used to delineate species boundaries [30, 59–64]. The Type Strain Genome Server, calculating a dDDH value of 79. % with the *X. euroxanthea* type strain, reports that NCPPB 4013 does not belong to *X. euroxanthea* and may represent a new species.

### *X. campestris* pv. *cannae* (* X. sacchari *)

The pathogen responsible for a bacterial leaf spot and leaf blight on canna (*Canna* x *generalis*) in India was described as *X. campestris* pv. *cannae* [65], though phylogenetic analysis of its *gyrB* gene sequence places the pathotype strain NCPPB 4345 in the *X. sacchari* clade [20]. Our phylogenomic analysis (Fig. 1) confirms a close relationship between NCPPB 4345 and the type strain of *X. sacchari *. However, the current species description for *X. sacchari* [16] states that ‘The strains of this species are isolated from diseased sugarcane’ and therefore would exclude this pathogen, which was isolated from canna rather than sugarcane. Currently, *X. sacchari* is not subdivided into pathovars, since there is no variation in the host range. This species is distinguished from other xanthomonads by its metabolic activity on a long list of carbon substrates [16], only a subset of which have been tested on *X. campestris* pv. *cannae*. Transfer of this pathovar into *X. sacchari* would require revision of its species description to encompass isolation hosts beyond sugarcane.

### *X. campestris* pv. *nigromaculans*

The causative agent of black spot on burdock (*Arctium lappa*) was originally described in 1927 under the name *Bacterium nigromaculans* [22] and subsequently renamed as *X. campestris* pv. *nigromaculans* [3]. Several authors have proposed, based on sequence-based phylogenetic analysis, that *X. campestris* pv. *nigromaculans* is more closely related to the species *X. hortorum* [6, 9, 10, 20, 21] than to the type strain of *X. campestris *. This proposal was initially based on just a single genetic locus, *gyrB* [20]. Later, Dia and colleagues arrived at the same conclusion on the basis of both MLSA and phylogenomic analysis of the core genome, but they did not make their *X. campestris* pv. *nigromaculans* genome sequence publicly available at that time [6], motivating our sequencing of its pathotype strain NCPPB 1935. Subsequently, Dia and colleagues publicly deposited their sequence data for this same strain [21]. Our phylogenomic analysis (Fig. 1) is consistent with the previous studies, placing this strain clearly into Dia’s sub-cluster A [6] within the *X. hortorum* clade, close to *X. hortorum* pv. *gardneri* and *X. hortorum* pv. *cynarae*.

Dia and colleagues [6] cite a report by Dehgan-Niri and Rahimian [66] when mentioning *X. campestris* pv. *nigromaculans*. That report identifies a leaf-spot pathogen of burdock (same host as pv. *nigromaculans*) to be ‘* X. gardneri *’, i.e. *X. hortorum* pv. *gardneri*, based on partial sequence of its *gyrB* gene [66]. Although Dehgan-Niri and Rahimian did not mention *X. campestris* pv. *nigromaculans*, their *gyrB* sequence shares 100 % nucleotide sequence identity with *X. campestris* pv. *nigromaculans* NCPPB 1935, as well as several *X. hortorum* strains; therefore, their burdock isolates are probably indeed pv. *nigromaculans*.

In summary, our genome sequencing and phylogenetic reconstruction supports previous suggestions that *X. campestris* pv. *nigromaculans* falls within a clade that corresponds to the species *X. hortorum* [6, 9, 10, 20, 21] and should be transferred to that species. We also note that our genome assembly for *X. campestris* pv. *nigromaculans* has a rather high level of contamination of 12.92 % whereas assembly GCA_938743425.1 [21] is of higher quality, with a contamination level of 0.81 % (Tables 1 and S1).

### Conclusion

Here we present draft-quality genome sequences for ten plant-pathogenic bacterial strains from the National Collection of Plant Pathogenic Bacteria (NCPPB). The data will be useful for phylogenomic studies, filling some important gaps in sequence coverage of the *Xanthomonas* phylogenetic diversity. Furthermore, these genome sequences may be useful in elucidating the molecular basis for important phenotypes such as biosynthesis of coronatine-related toxins and degradation of fungal toxin cercosporin.

## Supplementary Data

Supplementary material 1

Supplementary material 2
